# Comparison of QCT and DXA: Osteoporosis Detection Rates in Postmenopausal Women

**DOI:** 10.1155/2013/895474

**Published:** 2013-03-27

**Authors:** Na Li, Xin-min Li, Li Xu, Wei-jie Sun, Xiao-guang Cheng, Wei Tian

**Affiliations:** ^1^Department of Radiology, Beijing Jishuitan Hospital, No. 31 Xinjiekou Street, Xicheng District, Beijing 100035, China; ^2^Department of Spine Surgery, Beijing Jishuitan Hospital, No. 31 Xinjiekou Street, Xicheng District, Beijing 100035, China

## Abstract

*Objective*. To compare the osteoporosis detection rates in postmenopausal women when measuring bone mineral density (BMD) with quantitative computed tomography (QCT) in the spine versus dual X-ray absorptiometry (DXA) in the spine and hip and to investigate the reasons for the discrepancy between the two techniques. *Methods*. Spinal volumetric BMD was measured with QCT, and areal spinal and hip BMDs were measured with DXA in 140 postmenopausal women. We calculated the osteoporosis detection rate for the two methods. Lumbar CT images of patients who had a discrepancy between QCT and DXA findings were reviewed to evaluate vertebral fractures, spinal degeneration, and abdominal aortic calcification. *Results*. For the entire 140 patients, the detection rate was 17.1% for DXA and 46.4% for QCT, a significant difference (*P* < 0.01). Of the 41 patients with conflicting diagnoses, 7 whose diagnosis by QCT was osteoporosis had vertebral fractures even though their DXA findings did not indicate osteoporosis. Varying degrees of spinal degeneration were seen in all of the 41 patients. *Conclusion*. QCT may avoid the overestimation of BMD by DXA associated with spinal degeneration, abdominal aortic calcification, and other sclerotic lesions. It may be more sensitive than DXA for detecting osteoporosis in postmenopausal women.

## 1. Introduction

Osteoporosis is characterized by low bone mass and microarchitectural deterioration of bone tissue, leading to increased bone fragility and a consequent increase in fracture risk. Fractures may lead to a decreased quality of life and increased medical costs. Thus, osteoporosis is widely considered a major health concern.

Consequently, noninvasive techniques for measuring bone mineral density (BMD) play an important role in the clinical diagnosis of osteoporosis and in monitoring its progression. Dual X-ray absorptiometry (DXA) and quantitative computed tomography (QCT) are the most common tools for measuring BMD. DXA determines BMD in two dimensions, including both trabecular and cortical bone, with the results expressed as areal density (grams per square centimeter). QCT allows measurement of volumetric trabecular bone density without superimposition of cortical bone and other tissues, with the results expressed in milligrams per cubic centimeter of calcium hydroxyapatite.

DXA and QCT findings cannot be compared directly, and sometimes the diagnosis indicated by BMD findings differs between the two modalities. Therefore, we compared the detection rate of osteoporosis for posteroanterior DXAs (PA-DXA) with the rate for QCT and analyzed the reasons for the differences between the two.

## 2. Materials and Methods

Between February 2010 and February 2013, we reviewed data, for our study, for 194 postmenopausal women who underwent QCT and areal spinal and hip DXA in our department with an interval between QCT and DXA scans of two months. Study exclusion criteria included a history of multiple myeloma, rheumatoid arthritis, ankylosing spondylitis, systemic lupus erythematosus (SLE), connective tissue disease, metabolic and endocrine diseases, or bone tumors. Fifty-four patients were excluded, leaving 140 postmenopausal women as study participants.

DXA measurements were obtained using a Prodigy DXA scanner (GE, Lunar, Madison, WI, USA) and were analyzed using the manufacturer's software. The DXA T-score was calculated on the basis of the Chinese reference database [1]. Vertebrae from L1 to L4 and the left hip were scanned in the supine position using posteroanterior projections. The T-score for L1–L4 and for the femoral neck plus the total hip measurement by DXA were used to diagnose osteoporosis. We used the diagnostic criteria established by the World Health Organization (WHO) in 1994 [2].

QCT measurements were obtained with an Aquilion 64-slice CT scanner (Toshiba, Tokyo, Japan) with a solid Mindways QCT phantom (Mindways Software Inc., Austin, TX, USA). Vertebrae from L1 to L4 were scanned in the supine position. Images were analyzed using the Mindways software. Elliptical regions of interest were put in the midplane of three vertebral bodies (L2–L4) in the trabecular bone automatically, avoiding the cortical bone of the vertebrae. Fractured vertebrae were excluded from measurement. Average BMD is expressed in milligrams per cubic centimeter of calcium hydroxyapatite. For the BMD of spinal trabecular bone, thresholds of 120 mg/cm^3^ for osteopenia (equivalent to a DXA T-score of −1.0 SD) and 80 mg/cm^3^ for osteoporosis (equivalent to a DXA T-score of −2.5 SD) were suggested by the International Society for Clinical Densitometry in 2007 [3] and by the American College of Radiology in 2008 [4].

To estimate the degree of spinal degeneration and abdominal aortic calcification (AAC), two radiologists who were blinded to the BMD results independently reviewed lumbar CT images. The diagnosis in questionable cases was determined by consensus.

The difference between the osteoporosis detection rates for DXA versus QCT was analyzed using the chi-square test. Results were specified with a 95% confidence interval.

## 3. Results

The 140 study participants ranged in age from 47.1 to 85.9 years (mean: 63.2 ± 8.1 years). The interval time between DXA and QCT scans ranged from 0 to 43 days (mean: 4.3 ± 9.7 days). The BMD of L1–L4 as measured by DXA ranged from 0.575 to 1.621 g/cm^2^ (mean: 0.973 ± 0.169 g/cm^2^). The trabecular BMD of the lumbar spine as measured by QCT ranged from −5.0 to 199.4 mg/cm^3^ (mean: 81.7 ± 34.8 mg/cm^3^).

The osteoporosis detection rates for lumbar PA-DXA, lowest BMD of the femoral neck and total hip, lowest BMD for spinal and hip DXA, and lumbar QCT were 17.1%, 12.9%, 20.0%, and 46.4%, respectively (Table 1). The intragroup detection rates for QCT versus DXA were significantly different (*P* < 0.01), with QCT detecting osteoporosis more frequently than spinal and hip DXA, did.

**Figure 1:**
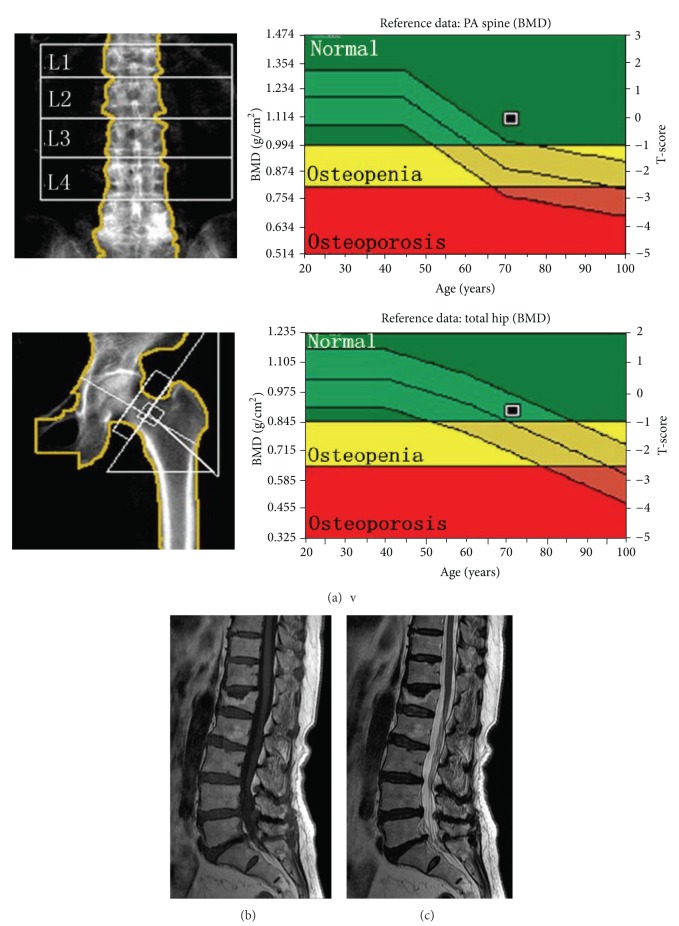
Images obtained from a 71.7-year-old woman whose bone mineral density was found to be normal on dual X-ray absorptiometry. (a) The T-scores for lumbar posteroanterior dual X-ray absorptiometry, the femoral neck, and the total hip were −0.1, −0.8, and −0.7, respectively. The trabecular bone mineral density of L2–L4 was 36.1 mg/cm^3^, and the diagnosis via lumbar quantitative computed tomography was osteoporosis. Sagittal lumbar spine images show two adjacent vertebral fractures with wedging, deformation of the end plates, and degenerative disc disease: (b) T_1_ weighted and (c) T_2_ weighted.

**Figure 2:**
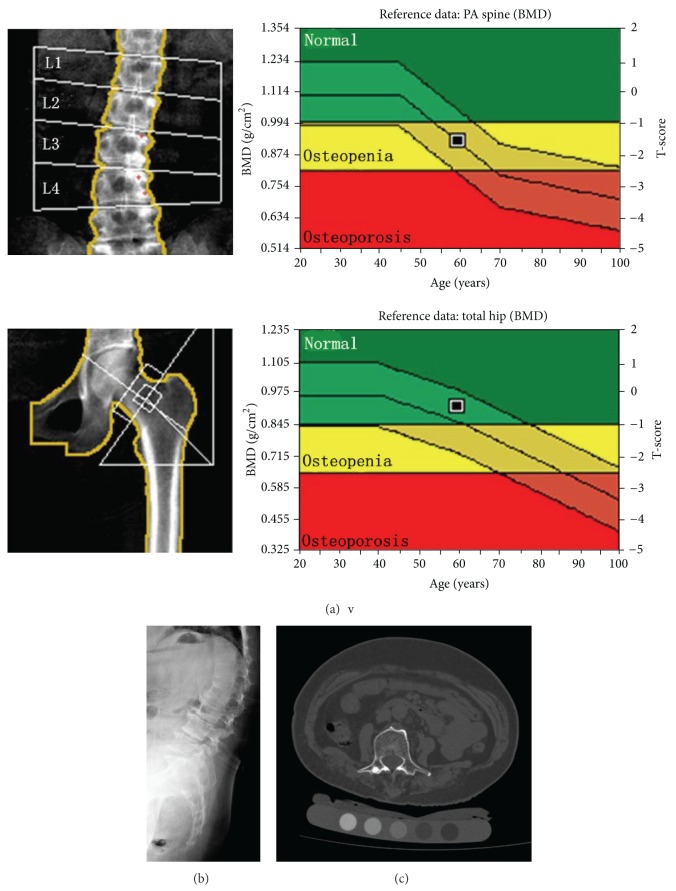
Images obtained from a 59.5-year-old woman who was found by dual X-ray absorptiometry to have osteopenia. (a) The T-scores for lumbar posteroanterior dual X-ray absorptiometry, the femoral neck, and total hip were −1.6, −0.5, and −0.4, respectively. The trabecular bone mineral density of L2–L4 was 71.4 mg/cm^3^, indicating a potential diagnosis of osteoporosis according to the American College of Radiology guidelines. A lateral lumbar radiograph (b) and an axial computed tomography image (c) showed severe osteophytes and end plate sclerosis of the lumbar vertebrae. Focal sclerosis is apparent on the right accessory of L3.

Of the 140 participants, 41 (29.3%) were found by QCT but not by DXA to have osteoporosis. Of these, 7 (17.1%) had single or multiple vertebral fractures (Figure 1). One of them underwent percutaneous vertebroplasty later. All 41 (100%) had vertebral osteophytes or end plate sclerosis, 33 (80.5%) had facet joint osteoarthritis, 14 (34.1%) had spinous process osteophytes, and 25 (61.0%) had AAC. In addition, 3 participants had bone islands or focal sclerosis (Figure 2). One participant had multiple calcifications in the abdomen.

## 4. Discussion

The diagnostic criteria for DXA established by WHO in 1994 have long been used as the gold standard in the clinical diagnosis of osteoporosis. The sites most commonly measured are the lumbar spine and hip. PA-DXA determines BMD in two dimensions. Spinal degeneration and AAC may lead to a false finding of increased BMD. By contrast, the site most commonly measured in QCT is the lumbar spine. Osteoporotic bone loss occurs mainly in trabecular bone. The turnover rate of trabecular bone is higher than that of cortical bone. Our study showed a significant difference in osteoporosis detection rates between DXA and QCT, providing clinical evidence that QCT has a greater diagnostic sensitivity than DXA.

Osteoporosis, spinal degeneration, and AAC are most commonly seen in the elderly, and the consequences of these conditions are more serious with increased age. This is why it can be problematic to use DXA rather than QCT; even though clinical findings may indicate osteoporosis, DXA may still indicate that BMD is normal. A possible explanation for the superior performance of QCT may be spinal degeneration and calcinations in the soft tissue around the spine. Yu et al. reported that BMD measured by PA-DXA was significantly higher in patients with spinal degenerative joint disease changes than in those without such changes, particularly when osteophytes were present at the vertebral bodies and facet joints [5]. Ito et al. indicated that the presence of osteophytes is associated with higher BMD when measured with DXA [6]. Our results show that spinal degeneration and AAC may be associated with the overestimation of BMD and the underestimation of osteoporosis by DXA. This may diminish the sensitivity of DXA for assessing osteoporosis.

Several clinical techniques for BMD measurement are available, including DXA, QCT, and ultrasound, each with its own advantages and shortcomings. Appropriate choice of technique and measurement site is important for the accurate diagnosis of osteoporosis. Schneider et al. found that, under the WHO classification, women with spinal osteoarthritis were more likely to be given a diagnosis of osteoporosis of the femoral neck and hip than those without spinal osteoarthritis but less likely to receive such a diagnosis when BMD was based on the PA spine (14.4% versus 24.5%). Schneider et al. recommended that, in women aged 65 years and older who are likely to have spinal osteoarthritis, DXA of the hip be used for identification of osteoporosis [7]. However, DXA of the hip still includes cortical bone, so, findings can be influenced by degenerative changes, leading to a decrease in the ability to detect osteoporosis. In our study, the detection rate of osteoporosis at any spinal or femoral site by DXA was significantly lower than the rate for QCT, and no femoral site was superior to the PA spinal site. QCT is truly a three-dimensional technique for quantifying BMD. It may measure BMD more accurately and reproducibly, especially in patients with spinal deformity, severe degenerative changes, extreme obesity, or low body mass index. QCT may be particularly useful in China, where DXA scanners are not available in most areas.

Greenspan et al. found that vertebral fractures were present in 18.3% of asymptomatic postmenopausal women and that 11.0% to 18.7% of individuals with clinical osteoporosis would have been classified as having normal bone by BMD criteria alone [8]. Ling et al. reported that vertebral fractures were present in 15% of women aged 50 years or older in Beijing [9]. In our study, we found vertebral fractures in 17.1% of women in whom QCT but not DXA showed osteoporosis. The extent of osteoporosis in these women may have been underestimated by DXA.

## 5. Conclusions

As our study demonstrated, QCT may avoid the overestimation of BMD by DXA associated with spinal degeneration, AAC, and other sclerosis lesions, such as bone islands. QCT may be more sensitive for detecting osteoporosis, but this must be validated in a larger population.

## Figures and Tables

**Table 1:** Diagnostic results of DXA versus QCT for 140 participants.

	Normal (%)	Osteopenia (%)	Osteoporosis (%)
Lumbar PA-DXA	69 (49.3)	47 (33.6)	24 (17.1)
Any femoral site by DXA	52 (37.1)	70 (50.0)	18 (12.9)
Any spinal or femoral site by DXA	40 (28.6)	72 (51.4)	28 (20.0)
Lumbar QCT	19 (13.6)	56 (40.0)	65 (46.4)

DXA: dual X-ray absorptiometry; PA-DXA: posteroanterior DXA; QCT: quantitative computed tomography.
